# Bioinformatics Approach to Mitigate Mislabeling in EU Seafood Market and Protect Consumer Health

**DOI:** 10.3390/ijerph18147497

**Published:** 2021-07-14

**Authors:** Gabriella Vindigni, Alfredo Pulvirenti, Salvatore Alaimo, Clara Monaco, Daniela Spina, Iuri Peri

**Affiliations:** 1 Department of Agriculture, Food and Environment (Di3A), University of Catania, via S. Sofia 100, 95123 Catania, Italy; clamonaco@unict.it (C.M.); daniela.spina@unict.it (D.S.); peri@unict.it (I.P.); 2 Bioinformatics Unit, Department of Clinical and Experimental Medicine, University of Catania, via S. Sofia 64, 95123 Catania, Italy; alfredo.pulvirenti@dmi.unict.it (A.P.); alaimos@dmi.unict.it (S.A.)

**Keywords:** food safety, common fishery policy, DNA barcoding, fish mislabeling, consumer information

## Abstract

Fisheries products are some of the most traded commodities world-wide and the potential for fraud is a serious concern. Fish fraud represents a threat to human health and poses serious concerns due to the consumption of toxins, highly allergenic species, contaminates or zoonotic parasites, which may be present in substituted fish. The substitution of more expensive fish by cheaper species, with similar morphological characteristics but different origins, reflects the need for greater transparency and traceability upon which which the security of the entire seafood value-chain depends. Even though EU regulations have made significant progress in consumer information by stringent labelling requirements, fraud is still widespread. Many molecular techniques such as DNA barcoding provide valuable support to enhance the Common Fisheries Policy (CFP) in the protection of consumer interests by unequivocally detecting any kind of fraud. This paper aims to highlight both the engagement of EU fishery policy and the opportunity offered by new biotechnology instruments to mitigate the growing fraud in the globalized fish market and to enforce the food security system to protect consumers’ health. In this paper, after a presentation of EU rules on fish labeling and a general overview on the current state of the global fish market, we discuss the public health implications and the opportunities offered by several techniques based on genetics, reporting a case study to show the efficacy of the DNA barcoding methodology in assessing fish traceability and identification, comparing different species of the *Epinephelus* genus, Mottled Grouper (*Mycteroperca rubra*) and Wreckfish (*Polyprion americanus*), often improperly sold with the commercial name of “grouper”.

## 1. Introduction

In recent years, the growth of the international fish trade has highlighted the need for improving the traceability of fishery products. A wide number of varieties of fish species are sold globally for human consumption, and therefore, consumers need clearer and more comprehensive information on fish labelling. Species substitution occurs in order to increase profits, substituting higher value species with other less desirable, often cheaper, less well-known or even illegal species [1]. Fish species substitution carries health risks for consumers, as illegal fish can enter the market without any sanitary checks [2]. In addition, species substitution can also occur accidentally when the species are quite similar morphologically, and thus the identities of species are easily mistaken. Concerns over mislabeling include purchasing less environmentally sustainable seafood; mislabeled food may generate negative impacts on marine populations, as it may lead to the consumption of products that come from poorly managed fisheries.

Correct labeling and additional precautionary measures are necessary, especially to preserve consumers’ rights with the aim of avoiding health problems derived from the consumption of species from contaminated areas and also for its possible ecological and toxicological consequences [3,4].

An informative label is necessary, especially for fishery products already pre-packed and prepared for consumption because the transformation and packaging processes tend to remove any recognizable external features [1]. However, when exposed to too much information, consumers could encounter an opposite problem: information overload causing indifference and misunderstanding [5].

The morphological identification of anatomical features of the macroscopic whole fish, using dichotomous keys published by the Food and Agriculture Organization (FAO) (Species Catalogues for Fishery Purposes) has for years been the sole method used to prevent fraudulent fish substitution [6]. However, it has been proven to be inadequate, especially in the case of processed fish products in which the morphological features have been removed. The need for high quality standards to guarantee public health and sustainability of fish market have raised new challenges in developing novel molecular traceability tools such as DNA barcoding, and other genetic markers linked to DNA. These tools support certification schemes and promote traceability already in force according to existing EU regulations. Genes are recognized as one of the three primary levels of biodiversity (along with species and ecosystems) and the incorporation of population diversity into management instruments and policies will aid the long-term sustainability of biodiversity [1].

Indeed, molecular approaches have several advantages such as high sensitivity (detection of few DNA molecules), DNA sequence diversity (also among phylogenetically similar species), good preservation and the resistance to food processing of nucleic acid materials [7]. It has been noted that DNA barcoding is used to accurately identify fish and fishery products using short unequivocal DNA sequences. For animals, the most promising gene is Citochrome Oxidase I (COI) [8].

The aim of this paper is to describe the opportunity presented by genetic techniques to strengthen the food security system and thereby protect consumers’ health. We first discuss the problem of fish substitution in the global market and the EU fishery policy to protect consumers. Then, we present the main health risks related to involuntary consumption of fish dangerous to consumers’ health. The methodology of the case study presented is focused on the genetic identification of the “grouper,” with the aim to find genetic differences from valuable species belonging to the *Epinephelus* genus and others of lower commercial value, which are improperly sold with the name “grouper.” This type of fraud is compounded by the fact that the grouper, like many other fish species, is very often sold as a prepared product in fillet form, thereby losing many of its morphological characteristics. Therefore, only the use of proper genetic markers (i.e., DNA Barcodes) allows their recognition and distinction. We built a phylogenetic tree using the Neighbor-Joining method (NJ) [9,10,11,12], which shows the evolutionary relationships of barcode sequences and gathered the different sequences according to the corresponding scientific names. This technique can be used to support the adoption of an integrated approach for a full implementation of the EU consumer fishery policy.

## 2. Frauds and Fish Species Substitutions

### 2.1. The Global Seafood Market

The demand for fishery products is rapidly growing worldwide. Presently, fishery products represent some of the most widely traded commodities. In parallel, due to globalization of the market, an increasing number of fish species are sold all over the world, creating confusion in consumers who encounter different species sold with the same commercial name [10]

According to the FAO [13], global fish consumption rose by 122%, from 1990 to 2018 with the annual per capita seafood consumption of fisheries and aquaculture products approximately double in 2018 compared to the level in the 1960s. The annual per capita consumption in the world has risen, from an average of 9.0 kg in 1961 to 20.3 kg in 2017, by live weight. Furthermore, Europeans consume on average 24.4 kg per person of fishery products annually, 4 kg more than the world average [14]. In the EU countries, the highest consumption is found in Portugal (56.8 kg) and the lowest in Hungary (4.8 kg). It is also interesting to point out that three quarters of the fish and seafood consumed in the EU comes from wild fisheries, while the remaining quarter comes from aquaculture. The Eastern European countries such as Romania, Bulgaria, Slovakia and Czech Republic have lower consumption due to their distance from the sea. The opposite case is seen in countries that border the sea, which make use of seafood products as one of their main sources of food. This is the case in Lithuania, Spain, France and Sweden. Among the prevalent species consumed in EU are tuna, salmon and cod.

**Figure 1:**
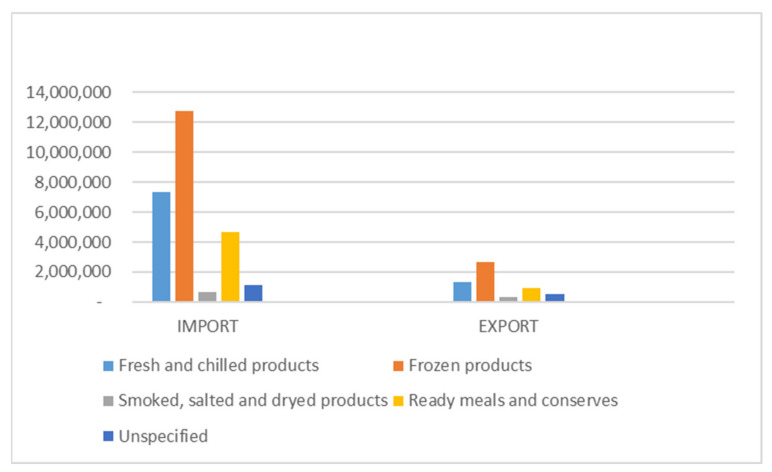
Imports and Exports of fishery and aquaculture products by main preservation categories—Extra EU-trade (value in thousands EUR). Source: our elaboration on European Commission data 2020.

The European Union is a net importer of fisheries and aquaculture products, mostly frozen, fresh and chilled, followed by ready meals and conserves and smoked, salted and dried, as shown in Figure 1. The most dynamic country is Spain, which has the highest level of both imports and exports to other countries, followed by Sweden, the United Kingdom and Denmark. In the global fishery market, Asia is the continent leader and China is the main fish producer and the most important exporter of fish and fishery products [14].

The fishing sector operates in an increasingly globalized environment: fish produced in one country can be processed in a second and consumed in a third. Many mislabeled products originate from small-scale retailers, convenience food producers and large organized distribution. Distributors and retailers, in order to increase their profit, tend to buy fish of lesser value, often cheaper and possibly injurious to human health or unfit for human consumption, rather than pay high prices for the fish stated on the label. The mislabeling of fisheries products generally arises after the products are bought from fisher folk, who must accept the market price.

Species substitution can arise at different stages throughout the seafood supply chain, especially in the case of rare, higher-priced species [15]. Fraud can occur in different contexts, such as restaurants, take-away, and foodservice companies. Especially worthy of note is the difference between fresh products and processed products, the latter of which are more difficult to detect as they lack morphological characteristics, making recognition nearly impossible [16]. Furthermore, processed fish is increasingly used as a raw material for novel products, such as protein powders, so the supply chain becomes even more nebulous and requires urgent changes for exactly identify fish species and improve traceability and food safety.

### 2.2. EU Fishery Policy and Pitfalls in Its Implementation

The Common Fisheries Policy has introduced new provisions to regulate labeling requirements about information on seafood to the consumer, which are applied in combination with those related to all European foodstuffs. The EU Regulation 1379/2013 has provided specific rules on consumer information for fishery and aquaculture products (FAPs), which have strengthened rules to prevent misleading practices and fraud. These provisions complement Regulation (EU) 1169/2011 on food information for consumers (FIC). Therefore, any aspect or product not covered by the common market organization (CMO) regulation is subject to the FIC regulation. Consequently, many consumers are more and more conscious of both nutritional and environmental issues, leading to a greater awareness regarding species, catch location and fishing methods [1].

CMO Regulation 1379/2013 (art. 35) legislates mandatory information on consumer labels for pre-packed and non-pre-packed, unprocessed and certain processed FAPs included in Annex I (points a, b, c and e), such as commercial designation, scientific name, production method and geographical area. In addition, it includes voluntary information (i.e., the date of catch or harvest, the type of fishing gear, the nutritional content and the production techniques). FIC regulation (art. 9 and 10) takes into account other information which must be displayed in the label (i.e., list of ingredients, allergenic ingredients, net quantity of the food, any special conditions for storage and use, date of expiration, name or business name, place of provenance and nutritional information). The FIC regulation also applies to processed FAPs not covered by Annex I of CMO regulation and, for this reason, the two regulations are complementary [2].

Both regulations act as tools to provide more information to consumers, however fish substitution fraud is not entirely prevented. 

**Figure 2:**
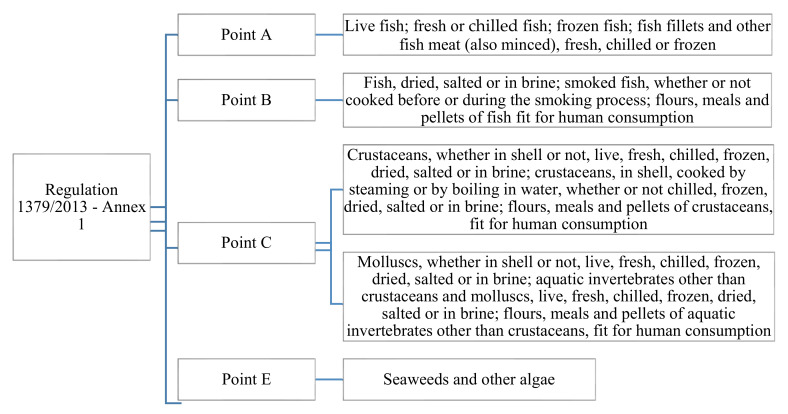
Processed FAPs covered by Annex 1, point a, b, c, and e of CMO Regulation 379/2013. Source: Our compilation from Regulation (EU) no 1379/2013 of the European Parliament and of the Council of 11 December 2013.

There is considerable debate about whether new labelling provisions for fish and fishery products can prevent seafood fraud from fish substitution. In EU countries, the practice of misleading labeling continues to be a serious problem despite the increasing importance of consumer information in EU legislation (Figure 2). 

The consumer information found on the labels of unprocessed or processed fresh products included in Annex I, point a, b, c, and e of the CMO regulation is summarized in the following table.

Even though both regulations act as a tool to prevent fraud by providing more information to consumers, there are some shortcomings in their implementation. The exclusion of processed FAPs from the art.35 (Annex I) can lead to misinformation [2]. Consumers require an understandable and complete label for processed FAPs, considering that convenience foods play an important role in fishery product marketing. Moreover, the legislator should establish clear indications for mass catering operators, which are not forced to display mandatory information in their menus. This is relevant as the percentage of mislabeling is 30% and usually fish substitution fraud occurs more in restaurants and takeaways than in retailers and supermarkets [17,18].

### 2.3. Public Health Implications of Fish Substitution

Fish fraud is usually perpetrated for financial gain. However, commercial fraud can also have serious consequences on consumers’ health [19]. Substituted fish can pose concerns due to the unintentional consumption of toxic and dangerous substances, highly allergenic species, pathogens and contaminants [20]. 

Zoonotic fish borne parasites in mislabeled fish represent a critical human health risk and inhibit proper diagnosis since the consumer is not aware of what they have ingested. Generally, through cooking and freezing it is possible to avoid parasite infections. However, the consumption of raw or inadequately cooked fish remains a significant risk factor. Concerns related to the consumption of mislabeled seafood are linked to allergies, contaminants such as mercury and other heavy metals and toxins [21]. Among these, tetrodotoxin and ciguatoxin are the most frequent and they may cause paralysis and potentially death if ingested in excessive quantities [22]. Tetrodotoxin occurs mainly in members of the family Tetraodontidae (puffer fish), which can replace monkfish. Another example of fish poisoning is due to ciguatera, which in the EU can be linked with the consumption of mislabeled imported tropical fishes, mostly snappers (*Lutjanidae*) and groupers (*Serranidae*) [4]. Another risk of poisoning due to contaminants is related to mercury and organochlorine compounds, which are considered carcinogenic and may lead also to negative neurobehavioral effects. Typical examples can be found in tuna and salmon which are often mislabeled. Additionally, one must consider that the complexity of the fish food chain is an ideal environment for those operators who want to implement fraudulent practices. 

Table 1 summarizes the main records collected globally regarding fish substitutions noticed at online vendors, retailers, restaurants, wholesalers, markets, groceries, and sushi restaurants [21,23,24].

## 3. Methods for Species Identification Using DNA Barcoding and SNPs

Molecular barcoding is currently the most commonly used methodology to verify if the species are correctly labeled and hence correspond with the label description [25,26,27]. This methodology, called DNA Barcoding, is not a new concept and its first use dates back to 1993. It consists of the utilization of short unequivocal DNA sequences to identify species. For most animals, the most promising gene is the Cytochrome Oxidase I (COI) gene [28]. In vertebrates, the Mitochondrial Cytochrome Oxidase (mtCOI) gene is 1545 bp long and is found within 648 bp of the beginning of the translated sequence known as the barcode. Different from molecular barcoding techniques used in the past, DNA Barcoding aims to standardize the process of identification into a single global system applied to a broad range of organisms. Fish represent the most relevant field of application [29]. Its principle is based on comparing the tag-sequence detected by molecular methods with the known sequences found in a web-database, thereby identifying the species by nucleotide sequence comparison [30]. An important feature of this method is the applicability to all life stages: because it is DNA-based it is irrelevant if it is applied at the stage of egg, adult or any other [31].

In addition, although it could be possible to detect a species by morphological methods, especially for fresh fish, in recent years several new species have been introduced on the market, which have been sold with common commercial names but with different scientific names. Therefore, in these cases, the detection is made possible only by molecular methods [32].

The Barcode of Life Data System (BOLD) represents the reference database where all species sequences are stored and available. Furthermore, the International Barcode of Life (iBOL) project has the ambition and the goal to barcode the world’s biodiversity. The Fish Barcode of Life (FISH-BOL) constitutes an important branch of research [33,34]. The success of the Barcode of Life project applied to fish products increased the use of its reference marker (COI) as a sequence-tag for fraud detection. Therefore, in recent years the majority of fish substitution studies were based on the COI sequence [35,36,37]. An interesting case is reported in research that confirmed the reliability of the DNA barcoding approach for seafood traceability and pointed out the Italian case of the genus *Mustelus*, especially for common smooth-hound (*Mustelus mustelus*). The percentage of commercial frauds of these species on the detected samples is high, up to 78%, even though only *M. mustelus* is clearly recognizable through the DNA barcoding approach whereas starry smooth-hound (*M. asterias*) is not discernible from other congeneric species. In any case, the proposed system has demonstrated high efficiency of the technique to discern *Mustelus spp.* from other [32].

An emerging class of genetic markers are the single nucleotide polymorphisms that consist of representing sites in the genome with minute mutations (novel genetic differences). Indeed, polymorphism derives from a genetic mutation where the type of polymorphism is typically referred to the specific mutation from which it has arisen. A single base mutation resulting from the substitution of one nucleotide for another is among the simplest possible types of polymorphism. In this case, the polymorphism at the changed site is named “Single Nucleotide Polymorphism (SNP)” [38].

SNPs are powerful molecular markers characterized by high polymorphism and reproducibility, and relevant density throughout the genome which are applied in phylogenetic analysis as well as in many other contexts like genetic diagnostics, population genetics, and mapping genes of interest. Hence, they represent a functional tool for dissecting complex traits via genome-wide association studies (GWAS) and quantitative trait locus (QTL) analysis [39,40]. SNPs have great importance as both markers of evolutionary history and in matching phenotypic traits with their genetic origins which makes them the largest source of genetic variation [41]. High density genotyping data such as those derived from SNPs analysis are indispensable for genomic evaluations of complex traits both in animal and plant species, where genotyping arrays aid the detection of associations between SNPs and phenotypes [39]. They are numerous, bi-allelic in nature as well as co-dominant and scattered along the genome. Many of them have also been identified in aquaculture species, being used as a genomic resource for developing a genetic linkage map for fish species such as Atlantic cod [42]. This represents a very actively developing area of research and commercialization [41].

SNPs are the most abundant type of DNA sequence polymorphism, whose variants have a significant role in genetic studies, featuring in the most widespread molecular markers [42]. On average, they can be found every 0.3–1 kilobases (kb) within the genome, which is the greatest known frequency of any type of polymorphism within the genome [37]). Efficient assays exist for genotyping, so the analyses of these very widespread SNPs reach high levels of population identification, making them suitable and optimal tools for seafood traceability [43]. In addition, results from SNPs exhibit high reproducibility between different laboratories. In fact, since their detection, the latest collected data can be compared with reference data, showing a high degree of reliability. Moreover, using SNPs as genetic markers allows users to identify changes unrelated to environmental differences or natural selection, so its detection permits users to distinguish features of local or regional groupings. The SNPs use is not as common as DNA Barcoding, but there is some evidence about its employment in the detection of fishery fraud. For example, in the Machado-Schiaffino et al. [43] research, the principal aim was to explore the extent of mislabeling in European hake markets, applying mitochondrial single nucleotide polymorphism (mtSNPs) methodology as a tool for rapid and accurate identification of hake species from the *Merluccius* genus. The method of mtSNPs proved to be highly reproducible, fast and technically easy to develop. It allows routine analysis of commercial seafood, and it does not require experts in genetics because both laboratory handling and interpretation of results are easy and direct. In the study a total of 40 commercially processed hake were analyzed and the results showed that 20% were mislabeled. Another more recent study [44] on European markets demonstrated that, by using gene associated single nucleotide polymorphisms, individual marine fish can be also traced back to population of origin with high levels of accuracy. This study was not based only on hake, but other three species were analyzed: cod, herring and sole. The results showed that the majority of individuals (between 93 and 100%) could be correctly assigned to population of origin by SNPs.

## 4. The Case Study of the Grouper Species Substitution in Italy

In this work we describe the grouper case study, as an example of common fish species substitution and the potential of the bioinformatics approach. The grouper belongs to the Serranidae family which includes over 500 species. More than thirty are listed among species of commercial interest approved by the Italian Ministry of Agricultural, Food and Forestry Policies (MIPAAF) according to the D.M. 27-3-2002 and following updates. The only species that can be sold with the commercial name of “grouper” in the label are: white grouper (*Epinephelus aeneus*), dogtooth grouper (*Epinephelus caninus*) and dusky grouper (*Epinephelus marginatus*). Furthermore, Polyprionidae as *Polyprion americanus* can be sold as grouper or wreck-fish. For all the other *Epinephelus* species, the MIPAAF clarifies that the label must specify a commercial name, for example “Dungat grouper” for the *Epinephelus goreensis*, “mottled grouper” for the *Mycteroperca rubra* and “spinycheek grouper” for the *Epinephelus diacanthus* [45].

*Epinephelus marginatus* and the *Polyprion americanus* are among the species with the largest presence and therefore the most targeted by professional and sport fishing activities. Some species are more at risk than others regarding fish stocks. The most threatened species is the dusky grouper that, even if common in many coastal waters [46], composes a Mediterranean subpopulation included as “Endangered” in the Red List of the International Union for Conservation of Nature (IUCN).

Regarding imports, the four main countries of origin for groupers in general are Argentina for Argentine seabass (*Acanthistius brasilianus*), Senegal for white grouper (*Epinephelus aeneus*), Vietnam for white grouper (*Epinephelus areolatus*) and South Korea for both brown-marbled grouper (*Epinephelus fuscoguttatus*) and white grouper [45]. As mentioned above, frauds mainly derive from processed and pre-packed food. Investigating the imports of 2014, 2015 and 2016 from the main countries of origin for groupers, a strong growth trend in imports from Vietnam can be seen, while imports from South Korea are declining.

In addition, further research detected 32% of mislabeling on all the species analyzed. The major frauds concerned the Mediterranean groupers substituted by the Nile tilapia (*Oreochromis niloticus*) a freshwater species also known as mango fish, native to Africa and often farmed in polluted waterbodies [6].

Cutarelli et al. [9] studied many seafood products, investigating if the species identified by morphological characters correspond with the results revealed by DNA methods. Results of sequencing corresponded with morphological identification for all the fish species analyzed except for the samples of the genus *Mullus* that were identified as species *M. barbatus* by sequencing and as species *M. surmuletus* by morphological investigation. This detection testifies that fraud of fresh products occurs mainly when they have a great commercial interest and present very similar morphological characteristics. According to these results, research by Meloni et al. [46] highlighted the same kind of fraud in the substitution of striped red mullet (*Mullus Surmuletus*) with other species of lower value like red mullet (*Mullus barbatus*) and West African goatfish (*Pseudupeneus prayensis*). The study also highlights cases of the European squid (*Loligo vulgaris*) and the Norway lobster (*Nephrops norvegicus*). It was detected that the European squid was substituted with southern shortfin squid (*Illex coindentii*) and other squids of the non-Mediterranean region, while northwest lobster (*Metanephrops australiensis*), Urugavian lobster (*Metanephrops rubellus*) and New Zealand lobster (*Metanephrops challengeri*) were marketed as Norway lobster. Due to the differentiation of the processed foodstuffs that characterizes the market, it is essential to develop and use tools for the unequivocal and quick identification of the species present in the market even when the morphological identification is no longer possible.

### 4.1. Methodology

In this section we report our application of DNA barcoding methodology on a segment of COI to compare fish sold under the commercial name of “Grouper” (*Epinephelus aeneus Epinephelus marginatus* and *Polyprion americanus*), with others subject to fraud (*Mycteroperca rubra*, *Epinephelus diacanthus* and *Oreochromis niloticus*). 

We used BOLD [47] to gather data on the COI sequences of each species (34 sequences for *Epinephelus aeneus*, 22 sequences for *Epinephelus diacanthus*, 64 sequences for *Epinephelus marginatus*, 12 sequences for *Mycteroperca rubra*, seven sequences for *Oreochromis niloticus*, 18 sequences for *Polyprion americanus* and 20 sequences for short-beaked common dolphin (*Delphinus delphis*), which was used as control) (Table 2). 

To analyze the relation among those sequences, we adopted the maximum likelihood method and Kimura two-parameter model [48] using the software environment MEGA X [49]. In Figure 2, we report the tree with the highest log likelihood (-8554.16). The robustness of internal branches distance was estimated by bootstrapping with 1000 replicates [50]. The percentage of trees in which the associated taxa clustered together is shown next to the branches. Initial trees for the heuristic search were obtained automatically by applying Neighbor-Join and BioNJ algorithms to a matrix of pairwise distances estimated using the Maximum Composite Likelihood (MCL) approach, and then selecting the topology with superior log likelihood value. This analysis involved 177 nucleotide sequences. There were a total of 1617 positions in the final dataset. 

### 4.2. Results

**Figure 3:**
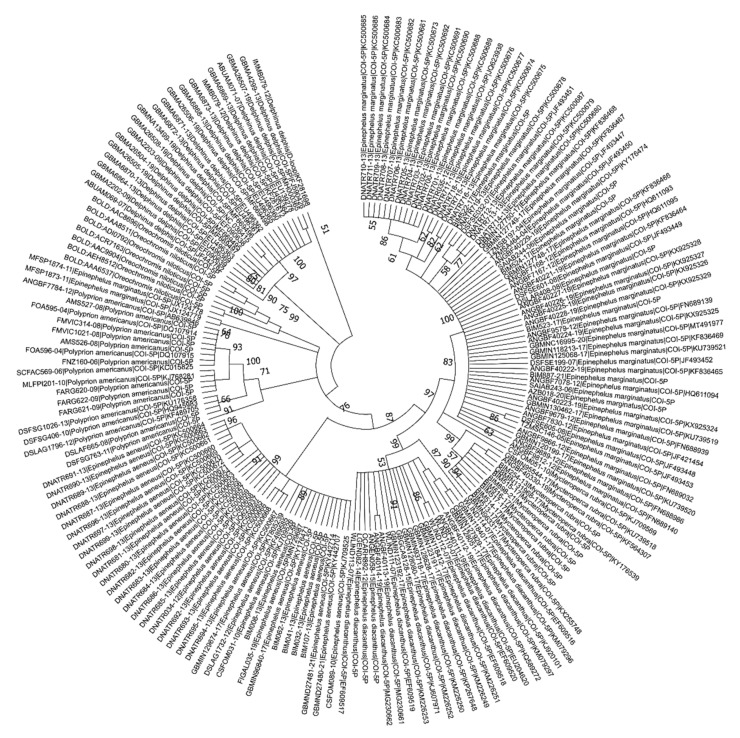
Evolutionary relationships of barcode sequences. Source: our compilation from BOLD data.

Our case study shows that the molecular identification of fish and processed fish products, through the COI Barcoding is effective (Figure 3). Furthermore, in some cases, the identification of the geographical origin of the specific fish (through the 5′Dloop) is also possible. This increases the knowledge on species of considerable importance for the Mediterranean area which are not yet well studied. The genetic analysis of the species of interest are exploited not only for the recognition of species–specific sequences, i.e., the ability to distinguish between two organisms belonging to different species, but also for determining the provenance of the fish (characterization of fish stock).

The evolutionary history was inferred by using the Maximum Likelihood method and Kimura two-parameter model. The tree with the highest log likelihood (−8554.16) is shown in Figure 3. The percentage of trees in which the associated taxa clustered together is shown next to the branches. Initial tree(s) for the heuristic search were obtained automatically by applying Neighbor-Join and BioNJ algorithms to a matrix of pairwise distances estimated using the Maximum Composite Likelihood (MCL) approach, and then selecting the topology with superior log likelihood value. The tree is drawn to scale, with branch lengths measured in the number of substitutions per site. This analysis involved 177 nucleotide sequences. There were a total of 1617 positions in the final dataset. Evolutionary analyses were conducted in MEGA X.

## 5. Conclusions

Political and consumers’ attention on the legal and health risks within seafood supply chains has grown. In this framework, sustainability in the fishing sector is linked to a shared traceability path aimed at protecting the consumer and the conservation of over-exploited natural resources.

Recent scientific advances, particularly in the fields of genetics and genomics, have led to the development of novel and improved technologies, and efforts are under way to harness their potential in this context.

The need for high quality standards to guarantee public health and the sustainability of fish markets has raised new challenges in developing new molecular traceability tools such as DNA barcoding and other genetic markers linked to DNA. These tools support certification schemes and promote traceability already in force according to the EU existing regulations. Genes are recognized as one of the three primary levels of biodiversity (along with species and ecosystems) and the incorporation of population diversity into management instruments and policies will further underpin an integrated approach to fisheries not only for the consumer, but also for long-term sustainability of biodiversity [1].

In a context so complex and globalized as the current seafood markets, the on-going need to ensure the high quality of fishery products for consumers has led to the development of many techniques for detecting different species, such as protein electrophoresis, restriction fragment length polymorphism (RFLP), amplification fragment length polymorphism (AFLP) and direct sequencing of specific genomic regions. Large-scale application is limited, in fact few research groups were able to use these techniques, each one on a case-by-case basis [25,26].

The phenomenon of globalization associated with the increased trade of seafood products has made DNA Barcoding one of the best ways to detect fraud occurring in the industrial food chain, since industrial processing systems make the detection more difficult, sometimes impossible, by classical identification approaches. Furthermore, DNA Barcoding shows high potential for replicability when applied to seafood products as well as to other animal sources because it is a DNA-based technique and its effectiveness has been confirmed by the accuracy of the results achieved in the majority of the studies. At the same time, the novel molecular methods based on SNPs could revolutionize origin assignment, becoming highly valuable tools for fighting illegal fishing and mislabeling worldwide due to their methodology (gene-associated markers). These methods permit users to go beyond the species level, allowing the identification of local varieties, and the origin of a certain product with low error rates [51,52]. Nevertheless, the SNPs methodology has to be implemented in order to standardize the process and to make the certification of the right origin of the seafood products easier. However, this case study has proved the efficiency of DNA barcoding to detect fraud between the grouper species and its capacity to include each sequence in the right cluster, showing also the genetic distance between the different species in terms of evolution.

Innovations such as product identifiers, that include DNA information or biochemical and geochemical signatures, could be relevant tools in the mitigation of fraud. However, these instruments are still insufficient to guarantee proper traceability in the fishing industry to protect consumers. To address these concerns, it is necessary to integrate rules and policies at the global level and at the same time consider the adoption of other tools, such as blockchain which is increasingly used in the agri-food sector. All this requires an accurate revision of the fish logistics and marketing systems and constant monitoring.

## Figures and Tables

**Table 1:** Examples of fish substitution in the world and associated consumer risks.

Declared Species	Substitute Species	Consumers Risks
Generic hake	Generic cod or pollock.	Presence of allergenics.
Generic cod	Olive flounder (*P. olivaceus*)	Presence of *Myxozoan* spp. if consumed raw.
Generic halibut	Generic flounder and Olive flounder	Not detected.
Escolar (*Lepidocybium flavobrunneum*)	Other generic escolar fish	Gastrointestinal illness, fat esters causing keriorrhea of varying exigency.
Generic snapper fish	Tilapia (*Oreochromis* spp.)	Ill-advised when consumed raw because of a wide range of fish-borne zoonotic trematodes.
Sablefish (*Anoplopoma fimbria*)	Patagonian and Antarctic toothfish	Species often infected with zoonotic species of *Anisakis* and *A. simplex*. Acute/chronic abdominal syndrome if consumed raw.
Atlantic cod (*G. morhua*), Pacific cod (*G. macrocephalus*), Black cod (*N. microlepidota*), Red rock cod (*S. papillosa*), Rock cod (*L. rhacina*)	Striped catfish (*P. hypophthalmus*)	*When consumed raw*, *probable presence of Haplorchis taichui*, *H. pumilio*, *Centrocestus formosanus.*
Common sole (*S. solea*), Wedge sole (*D. cuneata*), Winter flounder (*P. americanus*), Sand sole (*P. lascaris*), Senegalese sole (*S. senegalensis*), Oriental sole (*B. orientalis*)	Yellowbelly flounder (*R. leporina*), Largetooth flounder (*P. arsius*), Winter flounder (*P. americanus*), Greenback flounder (*R. tapirine*), European flounder (*P. flesus*).	*Probable presence of Anisakis* sp., *Hysterothylacium* spp., *P. decipiens*, *Contracaecum*, *H. aduncum* and *C. strumosum*.
Wreckfish (*P. americanus*), Grouper hybrid (*E. fuscoguttatus lanceolatus*), Hapuku wreckfish (*P. oxygeneios*)	Nile tilapia (*O. niloticus*), Tilapia (*Oreochromis* spp.).	Risk for fish-borne zoonotic parasites as *Gnathostoma*, *Cryptosporidium parvum*, *C. caninus*, *Clinostomum* sp., *Clonorchis sinensis*, *Contracaecum* spp., Cryptosporidium parvum, *Diphyllobothrium latum*, since often served raw at sushi restaurants.
Dactylopteridae or generic puffer fish	Silver-cheeked toadfish (*Lagocephalus sceleratus*)	Human illness, ciguatera, risk of death due to tetrodotoxin poison.
Farmed salmon	Wild caught salmon	Presence of organochlorine compounds which are considered carcinogenic and cause of negative neurobehavioral, immune or endocrine effects.
Swordfish (*Xiphias gladius*)	Shortfin mako (*Isurus oxyrinchus*), Porbeagle (*Lamna nasus*), Thresher sharks (*Alopias* spp.), Blue shark (*Prionace glauca*)	Not harmful but dangerous in case of allergy to the substitute species.
Sea bream (*Sparus aurata*), Common bass (*Dicentrarchus labrax*)	European seabass (*Dicentrarchus labrax*), Bluefish (*Pomatomus saltatrix*)	Not detected.
Atlantic bluefin tuna *(Thunnus thynnus*)	Yellowfin tuna *(Thunnus albacares*), Pacific bluefin tuna (*Thunnus orientalis*), Bigeye tuna *(Thunnus obesus*)	Not harmful but dangerous in case of allergy to the substitute species.
“Tobiko”/Flying fish egg (*Hirundichthys affinis*)	Bony flyingfish *(Hirundichthys oxycephalus*), Coromandel flyingfish (*Hirundichthys* *Coromandelensis*), Capelin (*Mallotus villosus*)	Not harmful but dangerous in case of allergy to the substitute species.
Sea bream (*Sparus aurata*)	Yellowtail amberjack (*Seriola lalandi*)	Not detected.
“Ikura”/salmon eggs	Chum salmon (*Oncorhynchus keta*)	Not harmful but dangerous in case of allergy to the substitute species

Source: Our elaboration.

**Table 2:** List of the species analyzed from BOLD. Linkage between species, corresponding accession number and source.

Scientific Name	Accession Number
*Epinephelus aeneus*	BIM008-13, BIM032-13, BIM041-13, BIM062-13, BIM107-13, CSFOM031-10, CSFOM089-10, DNATR034-12, DNATR680-13, DNATR681-13, DNATR682-13, DNATR683-13, DNATR684-13, DNATR685-13, DNATR686-13, DNATR687-13, DNATR688-13, DNATR689-13, DNATR690-13, DNATR691-13, DNATR692-13, DNATR693-13, DNATR694-13, DNATR695-13, DNATR696-13, DNATR697-13, DNATR698-13, DNATR699-13, DSLAG1732-12, FIGAL035-19, GBMIN129674-17, GBMIN96840-17, GBMND27480-21, GBMND27481-21
*Epinephelus diacanthus*	ANGBF40111-19, ANGBF40112-19, ANGBF40113-19, ANGBF40114-19, ANGEN058-15, GBGCA8128-15, GBMIN118401-17, GBMIN118412-17, GBMIN123191-17, GBMIN123192-17, GBMIN127929-17, GBMIN128051-17, GBMIN128080-17, GBMIN128628-17, GBMIN93718-17, LGEN082-14, OCARH892-12, WLIND110-07, WLIND111-07, WLIND112-07, WLIND113-07, WLIND114-07
*Epinephelus marginatus*	ANGBF40221-19, ANGBF40222-19, ANGBF40223-19, ANGBF40224-19, ANGBF40225-19, ANGBF40226-19, ANGBF40227-19, ANGBF40228-19, ANGBF40229-19, ANGBF7078-12, ANGBF7167-12, ANGBF7168-12, ANGBF7830-12, ANGBF9579-12, ANGBF9679-12, ANGBF9812-12, ANGBF9866-12, ANGBF9889-12, AZB018-20, BIM522-17, BIM523-17, BIM524-17, BIM887-21, DNATR035-12, DNATR700-13, DNATR701-13, DNATR702-13, DNATR703-13, DNATR704-13, DNATR705-13, DNATR706-13, DNATR707-13, DNATR708-13, DNATR709-13, DNATR710-13, DNATR711-13, DNATR712-13, DNATR713-13, DNATR714-13, DNATR715-13, DNATR716-13, DNATR717-13, DNATR718-13, DNATR719-13, DSFSE032-07, DSFSE199-07, DSFSE601-08, DSFSE605-08, DSLAR408-08, GBMIN118213-17, GBMIN120199-17, GBMIN125068-17, GBMIN127748-17, GBMIN127749-17, GBMIN127750-17, GBMIN130462-17, GBMNC16995-20, MFSP1873-11, MFSP1874-11, SAIAB243-06, SAIAB244-06, TZMSA464-04, TZMSB197-04, TZMSC146-05
*Mycteroperca rubra*	ANGBF40330-19, BIM254-13, BIM513-17, BIM514-17, BIM515-17, BIM516-17, BIM517-17, BIM898-21, CSFOM051-10, GBMIN131968-17, GBMIN95544-17, GBMIN96331-17
*Oreochromis niloticus*	AAC9904, AAA6537, AAA8511, AEH8512, AAC8696, ADI0792, ACR7163
*Polyprion americanus*	AMS526-08, AMS527-08, ANGBF7784-12, DSFSG1026-13, DSFSG406-10, DSFSG763-11, DSLAF665-08, DSLAG1796-12, FARG620-09, FARG621-09, FARG622-09, FMVIC1021-08, FMVIC314-08, FNZ160-06, FOA595-04, FOA596-04, MLFPI201-10, SCFAC569-06
*Delphinus delphis*	ABUAM069-07, ABUAM071-07, GBMA2202-09, GBMA2203-09, GBMA26504-19, GBMA26505-19, GBMA26506-19, GBMA26507-19, GBMA26508-19, GBMA4299-13, GBMA6864-13, GBMA6868-13, GBMA6869-13, GBMA6870-13, GBMA6871-13, GBMA6872-13, GBMA6873-13, GBMNA13499-19, IMMB079-12, IMMB079-12

## Data Availability

The data presented in this study are available on request from the corresponding author.
